# Exploring the therapeutic potential of *Emblica officinalis* natural compounds against hepatocellular carcinoma (HCC): a computational approach

**DOI:** 10.17179/excli2024-7970

**Published:** 2024-11-26

**Authors:** Sidra Ilyas, Abdul Manan, Yeojin Choi, Donghun Lee

**Affiliations:** 1 Department of Herbal Pharmacology, College of Korean Medicine, Gachon University, 1342 Seongnamdae-ro, Sujeong-gu, Seongnam-si, 13120, Korea; 2 Department of Molecular Science and Technology, Ajou University, Suwon 16499, Korea

**Keywords:** hepatocellular carcinoma (HCC), angiogenesis, docking, MD simulation, natural compounds

## Abstract

Hepatocellular carcinoma (HCC) is the fifth leading cause of cancer related deaths globally. Despite advancements in treatment, drug resistance and adverse side effects have spurred the search for novel therapeutic strategies. This study aimed to investigate how the *Emblica officinalis* can inhibit key targets involved in HCC progression. Screening of the reported compounds based on ADMET profile and identification of protein targets was done using the literature survey. Protein targets were divided into four major categories including inflammatory, angiogenic, anti-apoptotic as well as proliferative targets. Gene ontology (GO) and Kyoto Encyclopedia of Genes and Genomes (KEGG) analyses were performed to reveal the functional roles of genes. The STRING database was used to analyze the protein-protein interactions (PPI) of target genes. Docking was employed to predict the binding affinity of compounds with target proteins. Subsequently, MD simulation was conducted to assess the stability and dynamics of protein-ligand complexes. A total of 22 active compounds with 25 protein targets have been identified. These targets have a major role in controlling biological processes such as apoptosis, signaling and cellular interactions. KEGG pathway analysis showed that cancer, atherosclerosis, PI3K-Akt, EGFR tyrosine kinase inhibitor resistance and MAPK signaling pathways are mainly involved. Molecular docking by Mcule platform demonstrated that emblicanin A, punigluconin, penta-o-galloylglucose and quercetin showed higher binding energy affinities with BCL2, BCL2L1, c-Met, HSP70, EGFR, FGFR1, PTGS2 and TNFα. MD simulation revealed conformational changes, flexibility, interactions and compactness of protein-ligand complex. The stable protein binding interactions suggest the potential of compounds to inhibit the functions of target proteins. These results suggest that compounds derived from *E. officinalis* may have the therapeutic potential for treating HCC.

See also the graphical abstract(Fig. 1).

## Introduction

Hepatocellular carcinoma (HCC) is a rare primary liver cancer that has been associated with viral infections, alcohol intake, lipid accumulation and genetic/epigenetic alterations (Tian et al., 2022[44]). Nonalcoholic steatohepatitis (NASH), a disorder associated with hepatic inflammation and fibrosis, also termed as nonalcoholic fatty liver disease (NAFLD) is caused by fat accumulation in the liver (hepatic steatosis) (Berardo et al., 2020[6]). Moreover, prolonged and excessive alcohol intake can enhance simple steatosis to advance steatohepatitis and cirrhosis which can lead to development of alcoholic liver disease (ALD). Alcohol exacerbates liver inflammation, enhancing the translocation of endotoxins to the portal circulation by stimulating Kupffer cells to generate TNFα that causes hepatocyte damage via LPS and Toll like receptor 4 (TLR4) (Slevin et al., 2020[42]). Reactive oxygen species (ROS), ER stress, and the production of acetaldehyde are the three root causes of steatosis (Clare et al., 2022[12]). ROS inhibits the fatty acid oxidation (FAO) which is regulated by hepatic transcriptional factors such as AMP-activated kinase (AMPK) and peroxisome proliferator-activated receptor alpha (PPARα) (Alhayaza et al., 2020[3]; Teng et al., 2023[43]). In addition, gene mutations, chromatin remodeling and oxidative stress are the key drivers of Wnt/β-catenin and phosphatidylinositol 3-kinase (PI3K) signaling pathways.

Hepatocellular carcinoma (HCC) and cirrhosis developed from NASH and insulin resistance has resulted in malfunctioning of free fatty acids (FAAs) elimination pathways. Furthermore, transcription factors, PPARα and SREBP1c, can boost the synthesis of FAAs in the liver to activate important lipogenesis-related enzymes. Because of oxidative stress (OS) from ER stress and mitochondrial fatty acid β-oxidation, ROS generation is increased which in turn causes lipid peroxidation. ROS are responsible for DNA damage that may lead to the development of cancer (Ogunwobi et al., 2019[33]). Lipid peroxidation has the potential to trigger the release TNFα, IL8, TGF, and Fas ligand, which can lead to cell death, inflammation, and fibrosis. JNK activation triggered by TNF-α, FAAs, and ER stress have been linked to NAFLD progression and disease. A rise in JNK encourages the generation of cytokines and commences HCC.

Commonly used drugs for primary HCC include doxorubicin, cisplatin, fluorouracil, gemcitabine, capecitabine, and oxaliplatin; immunotherapy agent interferon-alpha, anti-angiogenesis agents such as bevacizumab and tyrosine kinase inhibitors such as sorafenib, sunitinib, erlotinib, and sirolinmus (Torimura and Iwamoto, 2022[45]). Neutralization and scavenging of free radicals by anti-oxidants from various medicinal plants may be responsible for liver cancer management and prevention. Medicinal plants (phytochemicals) act in various ways when applied to biological system and work in dose- as well as time-dependent manner. Medicinal plants are enriched in alkaloids, phenolic compounds, glycosides, peptides, terpenoids, nucleosides and flavonoids. Moreover, plant extracts have been reported as a crucial source of medicines for the management of liver disorders and drug discovery. One such medicinal plant has been extensively studied is *Emblica officinalis* (Indian gooseberry, family: Euphorbiaceae) rich in vitamin C, amino acids (proline, alanine, lysine, cystine, glutamic acid and aspartic acid), minerals and exhibit potent anti-oxidant, anti-inflammatory and anti-cancer properties (Deori et al., 2017[13]). Research studies have been revealed that it also contains alkaloids, phenolic compounds and tannins. Fresh pericarp contains higher concentrations of hydrolysable tannins including emblicanin A and B, pedunculagin and punigluconin. The fruit also contains ellagic acid, gallic acid, chebulagic acid, chebulinic acid as well as corilagin. A higher number of flavonoids like quercetin has also been reported (Bhandari and Kamdod, 2012[8]; Patel and Goyal, 2012[35]).

In this study, we aim to identify the potential key compounds from *E. officinalis* as inhibitors of key markers associated with HCC. Target proteins such as transcription factors, apoptotic proteins, growth factor receptors, cell division protein kinase and serine/threonine protein kinases that are involved in the pathogenesis of HCC were chosen. Through a systematic approach encompassing compound selection, target identification, and virtual screening, this research endeavors to contribute to the discovery of novel therapeutic strategies for the management of HCC.

## Experimental Procedures

### Collection of bioactive compounds of E. officinalis

An extensive literature search was carried out to identify the ligands that act as inhibitors for the protein targets mentioned. The compounds present in the fruit of *E. officinalis* that belong to different classes such as lignans, alkaloids, tannins, phenolics, coumarins and flavonoids were selected. A total of 22 compounds were chosen and their structures were downloaded from different small-compound databases including ChemSpider (https://www.chemspider.com/), PubChem (https://pubchem.ncbi.nlm.nih.gov/) and ZINC (https://zinc.docking.org/). 

### Target protein retrieval and preparation

**Figure 2:**
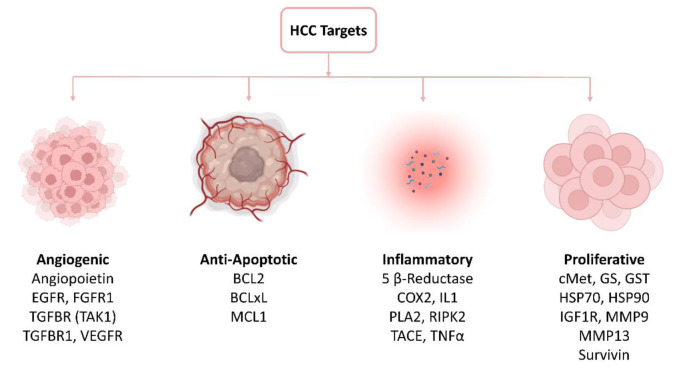
Classification of HCC protein targets

The crystal structures of target proteins reported to be associated with HCC were retrieved from the protein data bank (PDB). These proteins were divided into four groups including angiogenic, anti-apoptotic, inflammatory and proliferative protein targets (Figure 2(Fig. 2)). The amino acid residues linked with binding pockets for the inhibitor were also investigated.

### Drug-likeness and ADMET properties

Selected compounds were further analyzed by using Lipinski's rule of five (Ro5) and drug-likeness properties, compounds that showed Ro5 violations were eliminated (Lipinski, 2004[27]). To access the drug-likeness properties of compounds, ADMET properties (Absorption, distribution, metabolism, excretion and toxicity) were investigated by using FAF-Drugs3 tool. This serves for improving pharmacokinetic and pharmacodynamic properties of compounds prior to *in silico* modeling studies and screening protocols. FAF-Drugs3 facilitates pre-defined filters, but can be customized.

### Network construction and protein-protein interaction (PPI) analysis

The complex relationships between proteins and their roles in various biological processes were analyzed by STRING database (https://string-db.org/) and species confined to "Homo sapiens". A network of active compounds and HCC protein targets by using Cytoscape (version 3.10.0) was built in order to examine their interactions. 

### GO and KEGG enrichment analysis

Gene ontology (GO) and Kyoto Encyclopedia of Genes and Genomes (KEGG) pathway enrichment analysis were carried out on protein targets by using g-Profiler (https://biit.cs.ut.ee/gprofiler/gost). The GO terms were categorized into three types: cellular component (CC), biological process (BP), and molecular function (MF). The adjusted *p* ≤ 0.05 was utilized as the significance threshold.

### Molecular docking studies

Target proteins were docked against filtered 22 ligands using Mcule online server (https://mcule.com/), a drug-discovery platform. Mcule facilitates early-phase drug discovery by integrated computational capacity, high-quality compound database as well as molecular modeling tools. Mcule employs AutoDock program for molecular docking. The docking analysis facilitates binding ligand into the binding pocket of target protein and generates the best docked conformations with minimal energy. The ligands were kept flexible for docking while target proteins were kept rigid. The prepared proteins and selected ligand smiles were uploaded to an online drug discovery platform, mcule and the binding energies between the ligands and protein complexes were calculated. The docking results were evaluated using the Discovery Studio software by BIOVIA Discovery Studio (http://accelrys.com/products/) that provide 2D visualization of protein-ligand interaction. 

### Molecular dynamic (MD) simulations

Molecular dynamics (MD) simulations were employed to elucidate the dynamic behavior of the protein-ligand complexes. The CHARMM36 all-atoms force field was employed to model the docked complexes (Huang and MacKerell, 2013[21]), which were subsequently solvated in a cubic water box with a 12 Å buffer distance using the TIP3p water model. Ligand topology generation and compatibility with the Gromacs simulation package were achieved by CGenFF server and adapted for (https://cgenff.com/). System neutrality was ensured by adding appropriate numbers of Na^+^ and Cl^−^ ions. Energy minimization (steepest descent algorithm) was performed to a convergence criterion of 0.001 kJ/mol. Equilibration involves temperature and pressure control using the Nose-Hoover and Parrinello-Rahman methods, respectively (Bussi et al., 2007[10]). Long-range electrostatic interactions were treated using the particle mesh Ewald method whereas short-range electrostatic and van der Waals interactions were calculated within a cutoff radius of 1.2 nm (Essmann et al., 1995[15]). Production MD simulations of 200 ns were carried out for each complex using Gromacs 2024.1 software (https://www.gromacs.org/). Subsequent analysis of the resulting trajectories was done and structural visualizations were generated by using built-in tools of Gromacs. 

### Post-MD simulation data analysis and visualization

To elucidate the dynamics of protein-ligand complex, a comprehensive analysis of the generated MD trajectories was conducted. To assess the overall stability of the system, root mean square deviation (RMSD) calculations were performed. The flexibility of individual amino acid residues was evaluated by root mean square fluctuation (RMSF) which calculates the average displacement of each residue from its equilibrium position, thereby identifying regions of high flexibility with the protein. Furthermore, the radius of gyration (Rg) was computed to evaluate changes in protein compactness. Rg measures the mass distribution around the protein's center of mass, providing information about overall structural changes and potential folding/unfolding events. Hydrogen bond interactions (breakage/formation) between the protein-ligand was also analyzed throughout the 200 ns simulation.

## Results

### Bioactive compounds of E. officinalis 

The current study was to explore alternative inhibitory compounds present in *E. officinalis* against various HCC protein targets that may be helpful for better management of HCC. A total number of 22 compounds were selected from the literature reported for *E. officinalis*. The selected compounds were analyzed on the basis of physicochemical properties and Lipinski's rule of five (Ro5) to assess the drug-likeness (Table 1(Tab. 1)). Research revealed that emblicanin A, penta-o-galloylglucose, and punigluconin have shown unique features, good biological activity and possible therapeutic value for the treatment of HCC. Therefore, we have selected these compounds for docking studies although they violate Ro5.

### Protein targets of HCC

A comprehensive literature search was performed and protein targets (25) implicated in the pathogenesis of HCC have been identified and summarized in Table 2(Tab. 2).

### Network construction and PPI analysis

**Figure 3:**
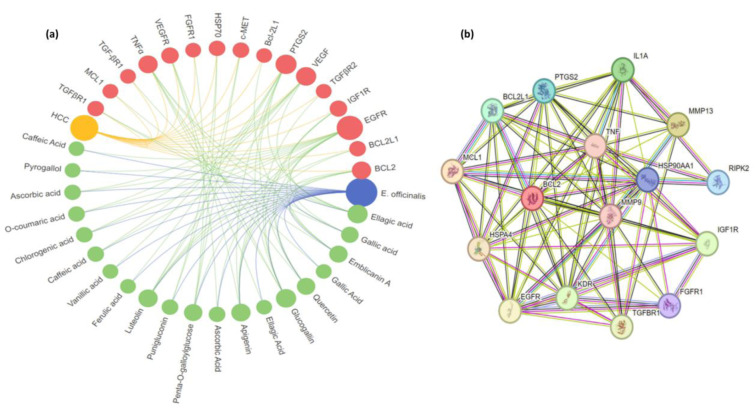
Network analysis of (a) compounds and target proteins where blue: herb, green: ingredient, red: gene and yellow: disease; (b) PPI network of proteins constructed by using STRING https://www.string-db.org/ database

For the treatment of HCC, the most significant and crucial active compounds were selected. Target proteins (16) were selected based on docking results and a network was constructed which reveals that numerous compounds can interact with a single/multiple HCC protein target and vice versa. The complexity of the interactions between protein targets and active compounds in *E. officinalis* is shown in Figure 3a(Fig. 3). The PPI interaction of the selected protein targets were also analyzed by STRING as shown in Figure 3b(Fig. 3).

### GO enrichment and KEGG analysis 

**Figure 4:**
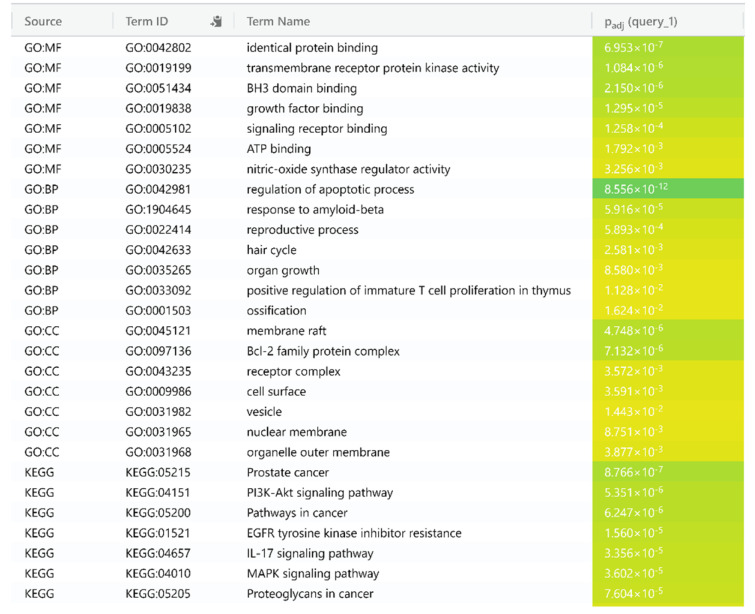
GO enrichment analysis and KEGG analysis of the protein targets for the treatment of HCC with *E. officinalis*

**Figure 5:**
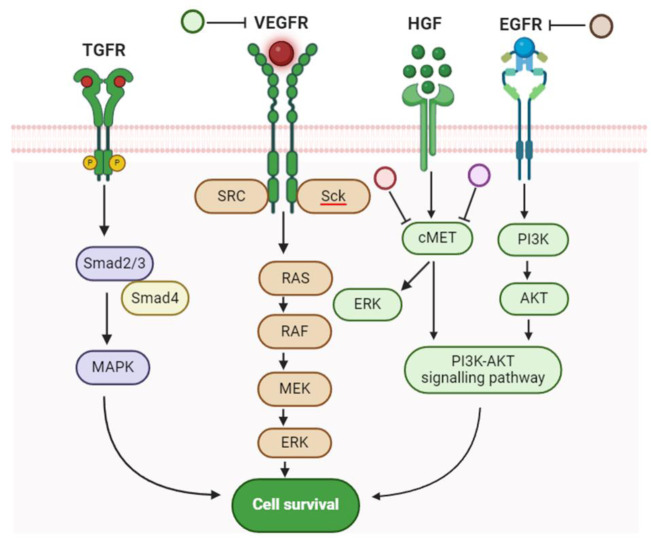
*E. officinalis *compounds such as emblicanin A (brown circle), penta-o-galloylglucose (purple circle), punigluconin (red circle) and quercetin (green circle) can target EGFR, c-Met, and VEGFR

GO enrichment analysis was performed on 16 HCC gene targets employing g:Profiler which showed 348 BP, 11 CC, and 35 MF terms were found that meet the screening threshold of *p* ≤ 0.05. The top 7 enrichment terms of BP, CC, and MF were presented in Figure 4(Fig. 4). The results of GO enrichment analysis indicated that the gene targets are implicated in multiple biological processes (BP) such as regulation of apoptotic processes, response to amyloid-beta, reproductive process, organ growth and T cell proliferation in thymus. In the enriched cellular component CC category, gene targets are implicated in the membrane raft, cell surface, BCL2 family protein and receptor complex. The enriched molecular function (MF) ontologies are dominated by identical protein binding, receptor kinase activity, growth factor binding and signaling. KEGG pathway analysis was performed on targets to elucidate the molecular mechanism by which *E. officinalis* treats HCC. A total of 34 enriched KEGG pathways were found to meet the screening threshold of *p* ≤ 0.05. The top 7 enriched KEGG pathways were presented in Figure 4(Fig. 4). The analysis showed that the molecular mechanisms by which *E. officinalis* treats HCC may be implicated in pathways in cancer, fluid shear stress and atherosclerosis, PI3K-Akt signaling pathway, EGFR tyrosine kinase inhibitor resistance, IL17 signaling and MAPK signaling pathway (Figure 5(Fig. 5)).

These signaling pathways could function together and modulating these pathways by *E. officinalis* compounds can have HCC therapeutic effects (Table 3(Tab. 3); References in Table 3: Ahn et al., 2019[2]; Ahmed et al., 2016[1]; Berdowska et al., 2021[7]; Chang et al., 2024[11]; Deori et al., 2017[13]; Hisaka et al., 2020[18]; Hussein et al., 2020[22]; Im et al., 2018[23]; Ji et al., 2019[24]; Kim et al., 2024[26]; Oh et al., 2001[34]; Ramadan et al., 2023[36]; Sajadian et al., 2016[38]; Shakerian et al., 2021[40]; Wei et al., 2011[47]; Yan et al., 2017[48]; Yang et al., 2018[50], 2021[51]; Zaazaa et al., 2018[52]).

### Molecular docking

**Figure 6:**
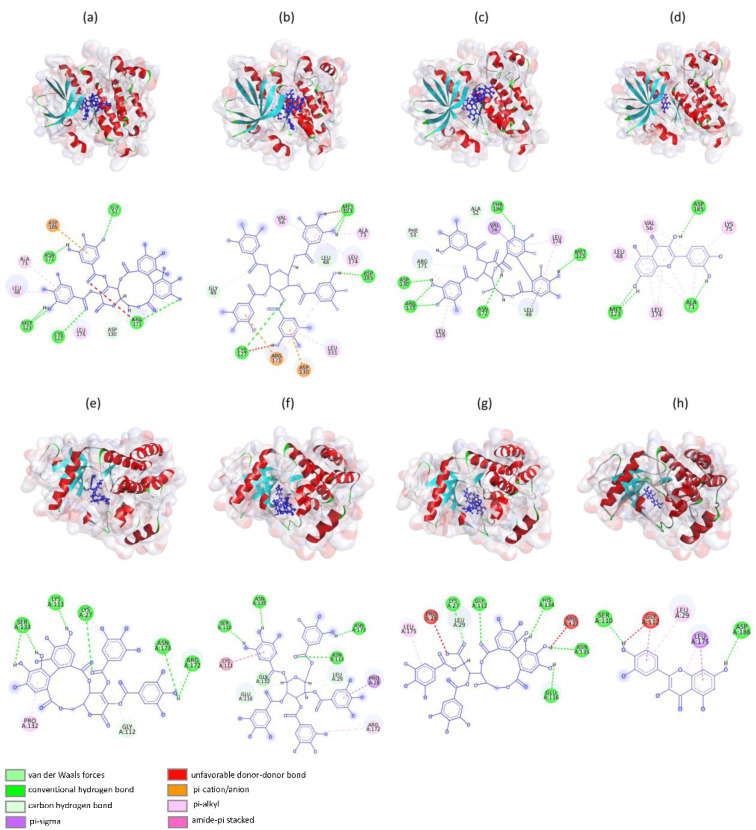
Molecular docking of HCC protein-ligand complexes and 2D and 3D visualization by Discovery Studio. Core targets EGFR with (a) emblicanin A, (b) penta-o-galloylglucose, (c) punigluconin, (d) quercetin and FGFR1 with (e) emblicanin A, (f) penta-o-galloylglucose, (g) punigluconin and, (h) quercetin

*E. officinalis* compounds have been docked *in silico* with HCC angiogenic, anti-apoptotic, inflammatory and proliferative protein targets to explore the best inhibitors that can be fruitful for the management of HCC. It can be seen from the heat map that a large number of compounds showed better binding affinity with HCC targets (Supplementary Table 1). Based on the heat map, the top four compounds emblicanin A, penta-o-galloylglucose, punigluconin, and quercetin that showed strong binding affinity have been selected against eight protein targets (BCL2, BCL2L1, c-Met, HSP70, EGFR, FGFR, PTGS2 and TNFα) shown in Figure 6(Fig. 6). Emblicanin A demonstrated highest binding affinity to Cox2, TNFα, c-Met with values of −10, −10, −9.7 kcal/mol. Punigluconin exhibited greater affinity with Hsp70, c-Met, FGFR with values of −10.2, −9.1 and −9.1 kcal/mol. The compound penta-o-galloylglucose showed greatest binding affinities with Hsp70, c-Met and TNFα with values of −10.5, −9.8 and −8.6 kcal/mol while quercetin displayed strong binding affinity values of −8.9, −8.0 and −9.1 kcal/mol with c-Met, Hsp90, and EGFR, respectively (Table 4(Tab. 4)). 

### Molecular interactions analysis

Drug binding location at its target is actually a key site for designing potential drug against any disorder. Docking results showed that compounds have been potentially involved in hydrogen bonding with polar residues including aspartic acid, histidine, serine and threonine. Hydrophobic residues such as leucine, valine, and alanine also contribute to the binding affinity through hydrophobic interactions. These interactions help to stabilize the protein-ligand complex and enhance binding specificity. Other interactions were also observed that enhance binding affinity of compounds with binding site (Table 5(Tab. 5)). The common amino acid shared by four compounds with EGFR include Leu174 and Ala73 that play crucial role in pi-alkyl interaction. Additionally, the amino acid Met123 interacts with all compounds with hydrogen bonds. In case of FGFR, Asn173 shared by both emblicanin A and penta-o-galloylglucose whereas Lys27 shared by emblicanin A and punigluconin have been involved in hydrogen bonding. Likewise, Ser110 shared by penta-o-galloylglucose and quercetin have been involved in hydrogen bonding. 

### MD simulation 

**Figure 7:**
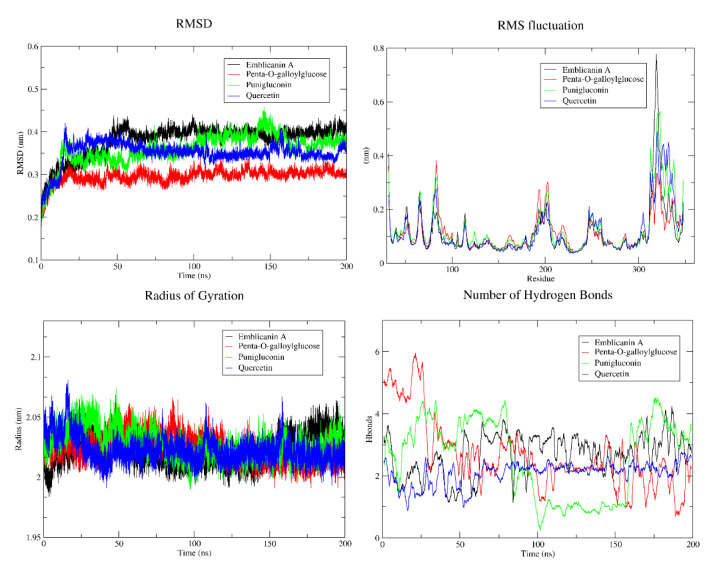
Molecular dynamics simulation of EGFR-ligand during the 200 ns time period. Analysis of RMSD, RMSF, Rg and hydrogen bond plots reveal insights into the structural stability, flexibility, compactness and intermolecular interactions

The RMSD and RMSF are the key metrics to analyze the conformation changes, structural stability, total fluctuation of individual amino acids and protein dynamics over time. The EGFR-ligand RMSD calculated during the 200 ns MD simulation fluctuated within a relatively narrow range, indicating protein maintained its overall conformation. EGFR-emblicanin A exhibited a higher RMSD plateau varying from 0.3 to 0.4 Å, indicating a higher degree of flexibility and confirmation changes, while EGFR-penta-o-galloylglucose showed a decreasing trend, suggesting a gradual convergence to a more stable confirmation over time. EGFR-punigluconin showed moderate plateau indicating intermediate level of structural flexibility when compared to emblicanin A and quercetin. A low RMSD plateau in EGFR-quercetin indicates relative stable structure with minimal conformational changes. The RMSF plot of emblicanin A at the amino acid residues (310-350) shows fluctuations that may be due to highly disordered protein regions of EGFR that exhibit considerable structural instability, flexible loops and hinges. EGFR-emblicanin A complex with higher degree of RMSF profile exhibited structural flexibility due to presence of disordered regions and intrinsic flexibility. EGFR-quercetin maintains a stable structure due to strong intramolecular interactions and rigid structure due to valine and isoleucine. Penta-o-galloylglucose with localized RMSF profile suggests specific region with higher flexibility. Low RMSF profiles of EFGR-punigluconin and EGFR-quercetin indicates more rigid structure. Higher Rg values of EGFR-emblicanin A indicates more extended and less compact structure. Intermediate values of penta-o-galloylglucose indicate moderately compact structure whereas a lower Rg in punigluconin and quercetin indicate a more compact and globular behavior of structures. A higher number of hydrogen bond formation was observed in both emblicanin A and penta-o-galloylglucose whereas a lower number of hydrogen bonds formation was seen in punigluconin and quercetin (Figure 7(Fig. 7)).

**Figure 8:**
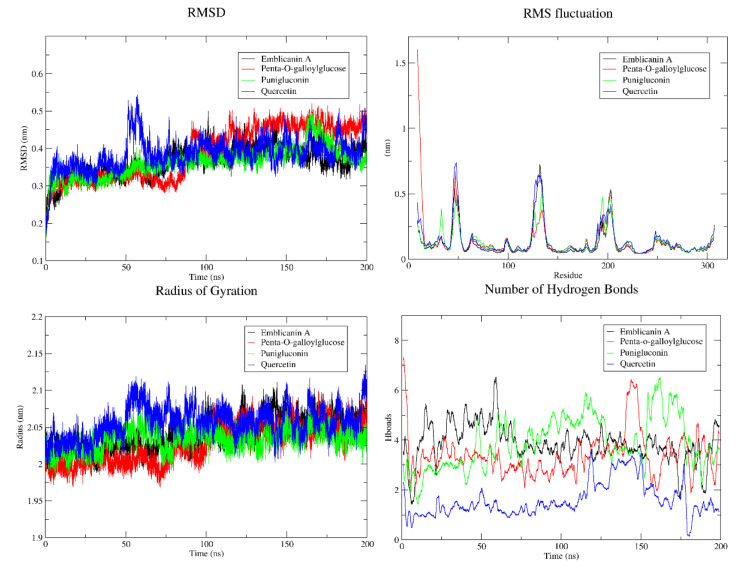
Molecular dynamics simulation of FGFR1-ligand during the 200 ns time period. Analysis of RMSD, RMSF, Rg and hydrogen bond plots reveal insights into the structural stability, flexibility, compactness and intermolecular interactions

FGFR-Penta-o-galloylglucose showed higher RMSD plateau from 0.3 to 0.5 Å as compared to emblicanin A, and punigluconin which indicates greater structural stability and conformational changes. A sudden high peak in RMSD indicates binding/release of quercetin that may be due to conformational changes in protein structure. RMSF plot of penta-o-galloylglucose show higher flexibility at amino acid residues (10-20) with Pro11/19, Arg15/20 at the beginning, and the amino acid residues at 40-70, 120-150, 190-220 may also be involved in interactions. Emblicanin A and quercetin also indicate higher RMSF profiles. A lower RMSF indicates rigid structure that helps to maintain overall stability of protein. A balance between flexibility and rigidity is required for overall functioning and stability of the protein. Rg (2-2.1 Å) was observed in case of all compounds, indicating that the binding of these ligands does not cause major conformational changes at the active site of the FGFR. A higher number of hydrogen bond formation was observed in all complexes, indicating a stronger propensity for the hydrogen bonding interaction (Figure 8(Fig. 8)). 

## Discussion

*E. officinalis* is widely used across several countries in South Asia and Southeast Asia including Bangladesh, Bhutan, Burma, China, India, Indonesia, Malay Peninsula, Malaysia, Pakistan, Sri Lanka, and Uzbekistan and commonly consumed for its reported benefits in treating liver and gallbladder diseases (Fatema Pria and Sayful Islam, 2019[16]; Kapoor et al., 2020[25]; Bhat et al., 2023[9]; Modi et al., 2023[32]). In China, various ethnic groups have distinct methods of using this plant, with some variations in preparation and application depending on local traditions and health benefits (Liu et al., 2024[30]). In this study, an *in silico* approach to address the inhibitory role of compounds of *E. officinalis* against various markers of HCC has been designed. *E. officinalis* consists of various compounds that are effective in treating HCC and we observed that the majority of these are multitarget. Research revealed that compounds present in *E. officinalis* attenuate oxidative stress in HCC by targeting proteins and signaling pathways (Sabir et al., 2022[37]; Asjad et al., 2023[5]). Flavonoids in *E. officinalis* such as apigenin and luteolin can inhibit HCC cell proliferation, migration, and invasion by inducing apoptosis via inhibiting the AKT/osteopontin and PI3K/Akt/mTOR pathways, respectively (Im et al., 2018[23]; Yang et al., 2018[50]). Phenolic compounds such as ferulic acid and vanillic acid have antioxidant properties and can target PTGS2 implicated in inflammation and tumor progression thereby inhibiting NF-κB signaling pathway (Kim et al., 2024[26]). Caffeic acid, chlorogenic acid, o-coumaric acid have been reported to exhibit anti-inflammatory and antioxidant properties that contribute to hepatoprotective effects by targeting tyrosine receptor kinases, EGFR and VEGFR, by modulating MAPK and PI3K/AKT pathways, respectively (Hussein et al., 2020[22]; Yang et al., 2021[49]). Likewise, pyrogallol inhibits PI3K/AKT/ Skp2/cMYC signaling pathways in HCC (Ahn et al., 2019[2]).

Tannins such as ellagic acid, gallic acid, and glucogallin have antioxidant as well as anti-inflammatory properties. These compounds can inhibit the HCC growth and proliferation via apoptosis by targeting inflammatory cytokine (TNFα) and tumor suppressor TGF-βR1 involved in NF-κB and SMAD signaling pathways, respectively (Ahmed et al., 2016[1]; Ramadan et al., 2023[36]). TNFα has tumor promoting/suppressing effects by stimulating/inhibiting angiogenesis and apoptosis (Ambade et al., 2016[4]). Ellagic acid has been shown to target STAT3 signaling pathway that is involved in tumorigenesis (Zaazaa et al., 2018[52]). Similarly, ascorbic acid (Vitamin C) could target proteins such as EGFR and VEGFR involved in oxidative stress and angiogenesis by targeting MAPK and PI3K/AKT pathways (Sajadian et al., 2016[38]). It can also attenuate oxidative stress in the liver by modulating immune responses (Wan et al., 2021[46]). Quercetin reduces growth of hepatic fibrogenesis in LX2 cell line by inhibiting TGF-β/Smad3 signaling pathways (Hisaka et al., 2020[18]; Shakerian et al., 2021[40]).

Key proteins (25) that play diverse roles in cellular processes, including HCC proliferation, survival, angiogenesis, and inflammation have been selected. Understanding their role can provide valuable insights into the disease's molecular mechanisms. GO and KEGG analysis revealed that genes are involved in diverse biological processes, primary related to signaling, cellular interactions and cancer-related pathways. Compound-protein interaction network analysis showed that emblicanin A, penta-o-galloylglucose, punigluconin and quercetin can target multiple proteins such as EGFR, FGFR1, BCL2 and Hsp90 mainly involved in HCC. PPI interaction showed that a high number of connections were observed among Hsp90, VEGF, EGFR and FGFR. Tyrosine receptor kinases (EGFR, FGFR1, IGF1R and VEGFR) often were overexpressed in HCC by promoting cell proliferation, survival, migration and angiogenesis and play crucial roles in activation of several signaling pathways (Shi et al., 2020[41]; Hu et al., 2021[20]). According to the Cancer Genome Atlas, HCC exhibited higher expression levels of FGFR and EGFR than normal tissues. Furthermore, EGFR and FGFR signaling is involved in a number of processes that lead to the development of HCC. The compound penta-o-galloylglucose can target both EGFR and FGFR1. TGF-βR1 plays a dual role, acting as a tumor-suppressor at early stages but contributes to tumor progression at later stages of HCC (Gonzalez-Sanchez et al., 2021[17]).

By preventing the release of cytochrome c and/or the binding of apoptosis activating factor (APAF1), the BCL2 family proteins (BCL2, BCL2L1, and MCL1) govern mitochondrial membrane permeability and apoptosis. Upregulation of the BCL2 family proteins in HCC promotes cell survival, prevents apoptosis thus contributes to tumor survival and growth (Liu et al., 2021[29]; Lv et al., 2022[31]). Apigenin and emblicanin A can induce apoptosis in HCC cells by inhibiting these anti-apoptotic proteins. Heat shock proteins (HSP70 and HSP90) are molecular chaperones that help maintain protein folding, stability and function. Their upregulation in HCC cells can contribute to tumor survival and resistance to chemotherapy, compounds in *E. officinalis* such as penta-o-galloylglucose and punigluconin can target both c-Met and Hsp70 (Liu et al., 2014[28]; Dong et al., 2018[14]). Similarly, PTGS2 can promote tumor growth, angiogenesis, and inflammation by generating lipid mediators from polyunsaturated fatty acids (PUFAs) via a lipoxygenase-type mechanism (Serhan et al., 2000[39]). Quercetin inhibits the increased expression PTGS2 and TNFα observed in HCC (Hu et al., 2023[19]).

Docking results revealed that all four compounds exhibited strong binding affinities to receptor tyrosine kinases signifying their possibility to inhibit cell proliferation and tumor growth. Emblicanin A and penta-o-galloylglucose have the highest binding affinities to antiapoptotic proteins (BCL2 and BCL2L1), suggesting their potential as promising anti-apoptotic agents. Notably, emblicanin A and penta-o-galloylglucose displayed highest binding affinities with tyrosine kinases, HSP70, and PTGS2, highlighting their potential inhibitory activity against cell proliferation, angiogenesis, and inflammation. Emblicanin A, penta-o-galloylglucose, punigluconin, and quercetin could potentially bind to EGFR/FGFR1, modulating their interaction by influencing EGFR and FGFR1 signaling. This could lead to changes in cell proliferation, survival, and differentiation. The interaction analysis showed formation of multiple hydrogen bonds between compounds and proteins which contributes to greater binding affinities suggesting therapeutic potential of *E. officinalis* for HCC treatment. 

By analyzing RMSD, RMSF, Rg and hydrogen bonding the conformational changes, flexibility, interactions and compactness of protein (EGFR and FGFR) with ligands (emblicanin A, penta-o-galloylglucose, punigluconin and quercetin) were explored. RMSD measures the overall deviation of protein's structure from initial state of structure and indicates overall stability of the complex whereas RMSF measures the average displacement of individual atoms from their equilibrium positions over time. The observed lower RMSD plot of EGFR-quercetin showed that protein is well folded and stable with minimal structural deviation under simulated conditions compared to other complexes. RMSF reveals local measure of flexibility of different region within protein complex to exhibit greater/lesser atomic motion. The higher peaks observed at various regions in RMSF were due to flexible amino acids (arginine, glucine, lysine, serine, and threonine) as compared to other rigid regions (valine and isoleucine). High RMSF values could be involved in protein-ligand binding. Radius of gyration (Rg) measures the average distance of atoms from a protein's center of mass to represent the compactness and rigidity of a complex as a function of time. Lower Rg values indicate more compact structure of protein-ligand complex as compared to more extending structure observed in higher Rg. Hydrogen bond formation suggests stronger intermolecular interactions and potential greater stability of the protein-ligand complex. The differences of hydrogen bond formation/breakage depend upon the specific amino acid residues, structural features of the protein and interaction of the surrounding environment. By integrating *in silico* approaches we identified promising natural compounds derived from *E. officinalis* capable of modulating critical molecular targets implicated in HCC pathogenesis and serve as potential hepatoprotective agents.

## Conclusions

The active compounds of *Emblica* *officinalis* showed promising potential for the treatment and management of hepatocellular carcinoma, as they exhibit versatile effects through their interaction with multiple protein targets and their involvement in multiple signaling pathways. Our comprehensive analysis identified that four compounds emblicanin A, punigluconin, penta-o-galloylglucose and quercetin can target eight proteins (BCL2, BCL2L1, c-Met, HSP70, EGFR, FGFR1, PTGS2 and TNFα) involved in multiple cellular processes and elucidating mechanism such as pathways, including cancer, PI3K-Akt, EGFR and MAPK signaling pathways associated with growth factors and receptors. Molecular docking and MD simulation studies further support the potential binding of these compounds to HCC related target proteins suggesting their direct therapeutic potential. While experimental validation is essential, our findings provide a foundation for developing novel HCC drugs derived from *E. officinalis* and offer valuable insights into therapeutic mechanisms. 

Although, computational approaches are powerful tools for drug discovery, they have inherent limitations in accurately predicting pharmacokinetics, pharmacodynamics and biological activity of the identified compounds. Additionally, *in vitro* and *in vivo* studies should be conducted to evaluate their efficacy and safety in animal models. Furthermore, incorporating additional computational techniques such as quantum mechanics calculations could provide deeper insights into potential off-target effects of the identified compounds. 

## Notes

Abdul Manan and Donghun Lee (Department of Herbal Pharmacology, College of Korean Medicine, Gachon University, 1342 Seongnamdae-ro, Sujeong-gu, Seongnam-si, 13120, Korea; E-mail: dlee@gachon.ac.kr) contributed equally as corresponding author.

## Declaration

### Conflicts of interest 

All authors declare that they have no conflict of interest.

### Authors' contribution

SI: Conceptualization, methodology, software, validation, formal analysis, investigation, data curation, writing - original draft preparation, visualization, project administration. AM: Methodology, validation, writing - original draft preparation, writing - review and editing. YC: Methodology, investigation, validation, writing - original draft preparation, DL: Writing - review and editing, project administration, funding acquisition.

### Funding

This research was supported by the Bio & Medical Technology Development Program of the National Research Foundation (NRF) funded by the Ministry of Science & ICT, Korea (grant number: 2020M3A9E4104380).

## Supplementary Material

Supplementary information

## Figures and Tables

**Table 1:**
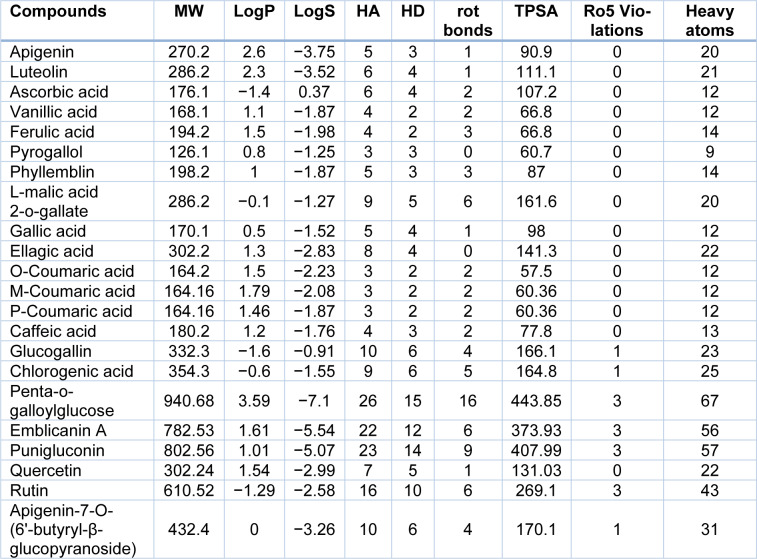
Predicted drug-likeness properties of potential compounds from *E. officinalis*

**Table 2:**
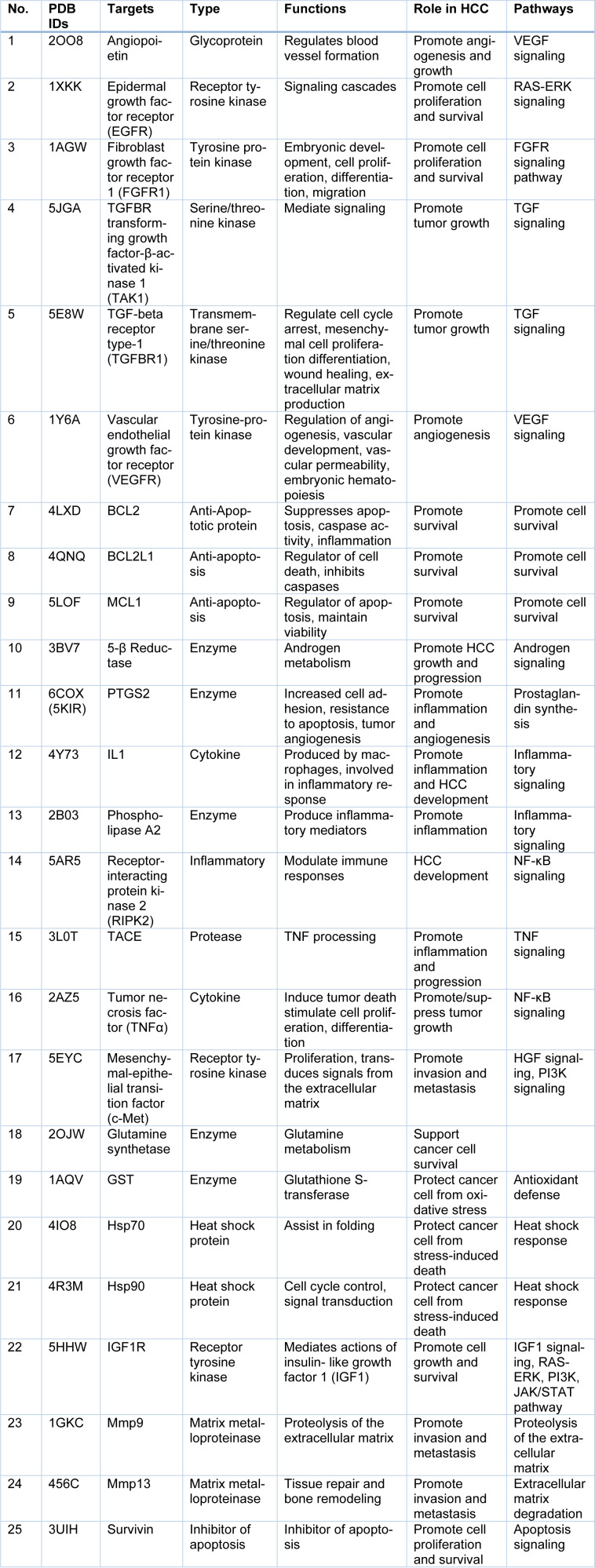
List of HCC protein targets with their PDB IDs and biological significance

**Table 3:**
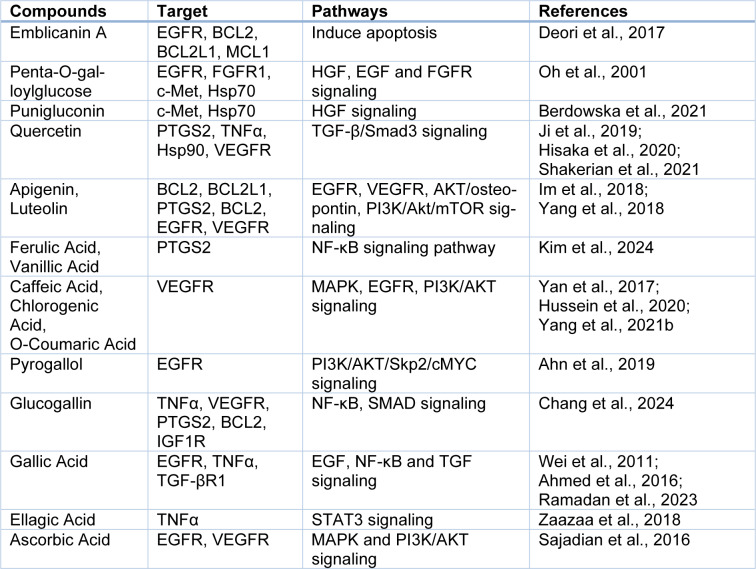
*E. officinalis* compounds with multiple protein targets with pathways involved in HCC

**Table 4:**
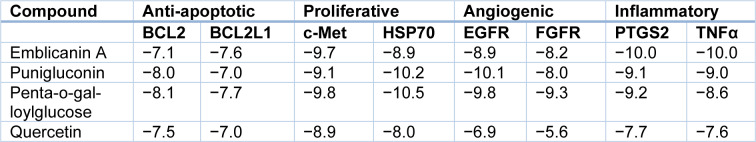
Top compounds of *E. officinalis* with binding affinities (kcal/mol) selected for MD Simulations

**Table 5:**
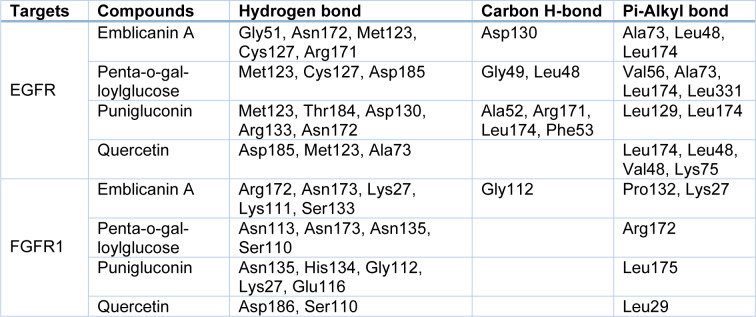
Protein-ligand interactions of EGFR and FGFR1

**Figure 1:**
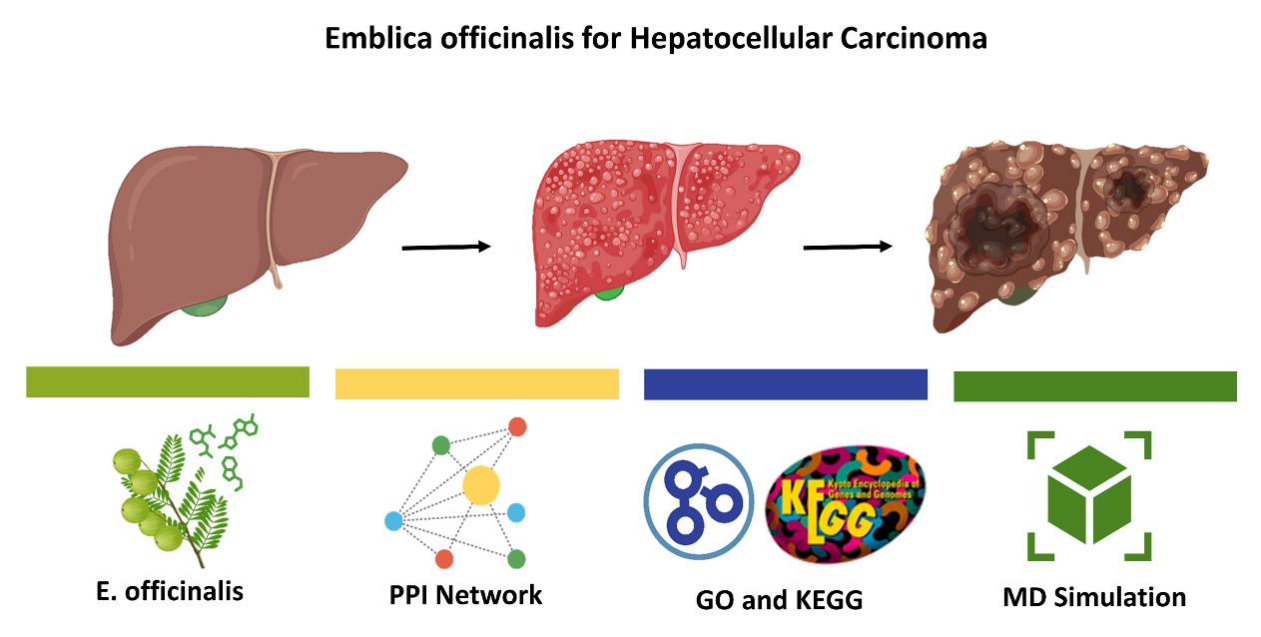
Graphical abstract
